# Tissue alkalosis in cold-ischemia time

**DOI:** 10.1038/s41598-017-11284-z

**Published:** 2017-09-07

**Authors:** V. Denninghoff, E. H. R. Olivieri, C. Fresno, A. Uceda, L. Mota, A. P. Suenaga, D. M. Carraro, V. R. Martins, A. Avagnina, F. A. Soares, A. H. J. Fróes Marques Campos

**Affiliations:** 1 Department of Pathology, A. C. Camargo Cancer Center, São Paulo, Brazil; 2 Department of Pathology & Biobank, Center for Medical Education and Clinical Research “Norberto Quirno” (CEMIC), Ciudad Autónoma de Buenos Aires, Buenos Aires, Argentina; 3 International Center for Research (CIPE), A. C. Camargo Cancer Center, São Paulo, Brazil; 4 A. C. Camargo Biobank, A. C. Camargo Cancer Center, São Paulo, Brazil; 5 Computational Genomics Division, National Institute of Genomic Medicine (INMEGEN), México city, Mexico

## Abstract

The control of pre-analytical-factors in human biospecimens collected for health research is currently required. Only two previous reports using post-mortem brain samples have tried to address the impact of cold-ischemia on tissue pH. Here we report pH variations according to time (third-order polynomial model) in mice for liver, kidney and lung samples. Tissue alkalosis in cold-ischemia time may be an underlying mechanism of gene expression changes. Therefore, tissue-pH regulation after organ removal may minimize biological stress in human tissue samples.

## Introduction

Biospecimens collected by tissue banks may provide valuable insights into the mechanisms of a wide range of diseases, thus leading to the discovery of biomarkers of diagnosis, prognosis and therapeutic guidance^1–3^. There is a consensus that high-quality, data-rich samples are essential for future research; however, their collection and storage is not as straightforward as many researchers think^3^. When it comes to transcriptional analysis, this concern is especially true, since gene expression can be modulated by various factors^4^. In terms of high-throughput transcriptional analysis, the highest RNA quality is recommended. However, the real impact of tissue specimens’ pre-analytical factors on the transcriptional regulation, besides the RNA integrity, is not well understood. While technical issues such as preparation, quality and concentration of RNA are of great concern for the use of analytical tools and platforms, those related to tissue collection, such as cold ischemia, have been recently considered^5–10^. An increasing number of reports have demonstrated that inadequate human biospecimen handling can have an adverse impact on assay results and biomarker discovery, with a negative effect on the clinical practice. However, the underlying mechanism leading to such changes in gene expression is not wholly defined^11, 12^. The RNA Integrity Number (RIN), a measurement of ribosomal RNA integrity, has been used to evaluate the overall RNA quality in biospecimens^13^. Additionally, RIN measurement fails to reflect the influence of the external factors that may have an impact on the modulation of gene expression, while not interfering with the overall RNA degradation. Specific and uncertain surgical factors, such as warm-ischemia time, are hard to be controlled. Moreover, the length of time a tissue sample could stay at room temperature after excision and before freezing is currently a matter of debate. The impact of cold-ischemia time may possibly vary by organ, with some of the organs requiring a much faster freezing process^3, 14^. In order to adequately investigate the underlying mechanism involved, a strict control of tissue ischemia times before cryopreservation is also required.

Mice are the vertebrates most widely used in scientific research, due to their genetic similarities with men. Approximately 99% of the human genes have been mapped into mice, making these vertebrates suitable for comparative studies^15^. In addition, working with neoplastic disease-free mice allows avoiding the introduction of disease variables, which could not be bypassed with cancer patients’ tissues. Finally, a quality control marker identified in an animal model, particularly in mice, may be applied to human biospecimens, provided that the corresponding molecules or pathways are represented in this animal model^11^. Therefore, we investigated the impact of devitalization on tissue quality using a mouse model, to find out its effect of pH and RNA integrity after a cold ischemia insult.

## Results

**Figure 1:**
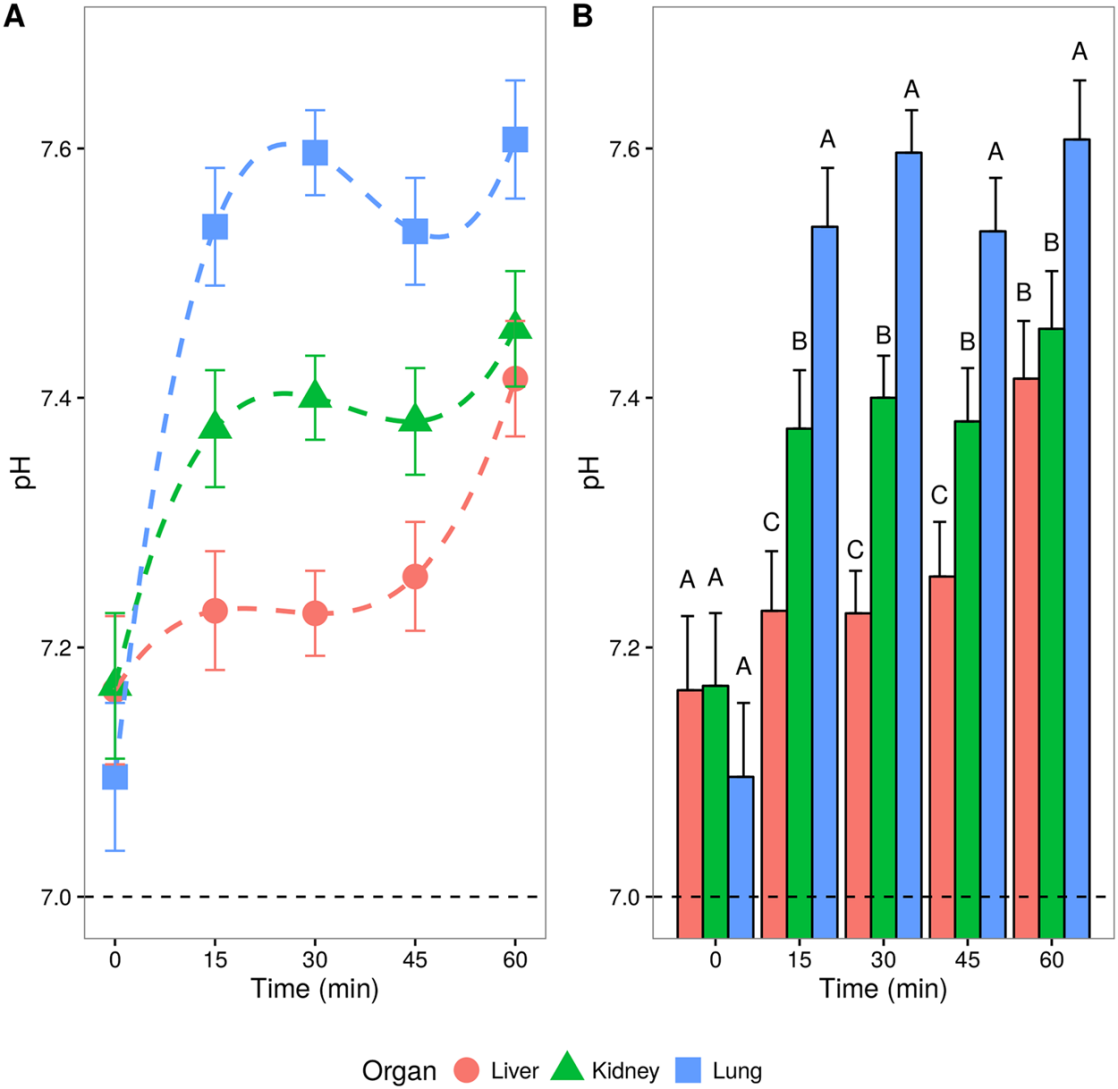
pH variation  according to ischemia time evolution in three different organs. (**A**) Organs third-degree adjusted time polynomial linear mixed model results (dashed lines). (**B**) Fisher’s least significant difference between organs mean at each data point. Superscript letters indicate significant difference (p < 0.05) using Bonferroni adjusted p-values. In both panels results are presented as mean ± standard error estimation.

Mice were euthanized and the pHs of kidney, liver and lung samples were measured at 0, 15, 30, 45 and 60 minutes after stopping the blood flow^16^. This time interval was chosen considering the maximum acceptable cold ischemia time reported in the literature^17^. Complete model description and results are described in equations (1–9) and Supplementary Table S1. A pH significant increase according to time (third-order polynomial model) was found in mice for liver, kidney and lung (Table 1). Fisher’s least significant difference remained unchanged at time-zero (Fig. 1). Following the ischemic insult, time dependence was always higher in lung, followed by kidney and liver, with a pH range of 0.64, 0.4 and 0.36, respectively. Although RIN’s statistical power did fit a second-order time evolution (Supplementary Fig. S5), in biological terms, this result does not modify its clinical use. In this study, the adjusted RIN was greater than 8.90, with the minimum acceptance threshold being RIN = 7. On the other hand, organ histoarchitecture (data not shown) and RNA absorbance ratios remained unchanged over the time for each organ (Table 2). Supplementary data is available at Scientific Reports’ website (Supplementary Table S1 and Figs S1–S6).

**Table 1:** Ischemia time dependent variables.

		Organ	N	Time (min)					Interval
				0	15	30	45	60	
Variable	pH	Lung	200	7.09 ± 0.06^A^	7.54 ± 0.05^A^	7.60 ± 0.03^A^	7.54 ± 0.04^A^	7.62 ± 0.05 ^A^	[7.03, 7.67]
		Kidney	206	7.16 ± 0.06^A^	7.37 ± 0.05^B^	7.40 ± 0.03^B^	7.37 ± 0.04^B^	7.45 ± 0.05^B^	[7.10, 7.50]
		Liver	204	7.17 ± 0.06^A^	7.24 ± 0.05^C^	7.23 ± 0.03^C^	7.26 ± 0.04^C^	7.42 ± 0.05^B^	[7.11, 7.47]
	RIN	Lung	100	9.04 ± 0.14^C^	9.34 ± 0.11^B^	9.45 ± 0.11^A^	9.37 ± 0.11^A^	9.11 ± 0.18^B^	[8.90, 9.56]
		Kidney	103	9.99 ± 0.13^A^	9.67 ± 0.11^A^	9.42 ± 0.11^A^	9.23 ± 0.11^A^	9.11 ± 0.12^B^	[8.99, 10.00]
		Liver	102	9.45 ± 0.14^B^	9.34 ± 0.11^B^	9.32 ± 0.12^A^	9.39 ± 0.12^A^	9.55 ± 0.12^A^	[9.20, 9.67]

Model results are expressed as mean ± standard error for each variable of interest. Different superscript letters indicate Fisher’s least significant difference (p < 0.05) using Bonferroni adjusted p-values for mean comparison. N: number of sample. RIN: RNA integrity number.

**Table 2:** Ischemia time independent variables.

Organ	RNA		Organ relative weight		
	260/280	260/230	Total	Right	Left
Lung	2.11 ± 0.01^B^	2.09 ± 0.01^B^	0.64 ± 0.004^C^	0.43 ± 0.01^A^	0.20 ± 0.01^B^
Kidney	2.13 ± 0.01^A^	2.19 ± 0.01^A^	1.34 ± 0.004^B^	0.69 ± 0.01^A^	0.65 ± 0.01^B^
Liver	2.10 ± 0.01^B^	2.12 ± 0.01^B^	4.64 ± 0.10^A^	—	—

Model results are expressed as mean ± standard error for each variable of interest. Different superscript letters indicate Fisher’s least significant difference (p < 0.05) using Bonferroni adjusted p-values for mean comparison. Note that “—” indicates result not available for the organ. RNA 260/280 and 260/230 are the RNA absorbance ratios at 260 nm/280 nm and 260 nm/230 nm, respectively.

## Discussion

Two phenomena must be highlighted for a better understanding of our work: on the one hand, chronic pH profile after brain ischemia evidences the duration and presumably the severity of ischemic injury. After the onset of stroke symptoms, intracellular pH has been found to increase up to 0.45 pH units in regions of cerebral infarction. Brain tissue alkalosis cannot be attributed to abnormal blood gases, because post-ischemic blood gases were found to be within normal physiological ranges. Whether alkalosis is secondary to ischemic cell damage, or it may contribute to ischemic cell damage, remains to be determined^18^. In their study, Chopp *et al*. described a 0.45 pH units variation. We could observe a 0.64, 0.40, and 0.36 pH units variation, between 0–60 minutes for lung, kidney, and liver, respectively, with an interval of [7.03, 7.67], [7.10, 7.50], and [7.11, 7.47] (Table 2). There is no previous evidence of tissue pH measurement in tissues submitted to ischemia, except for the brain. Therefore, this study is novel in the way it looks at tissue pH following cold ischemia insult, and because it explores this phenomenon in three different organs. As outlined in the work of Chopp *et al*., and according to our data, we would hypothesize that tissue pH measurement varies substantially, regardless of blood pH^18^. Unfortunately, in the experimental design performed herein we were unable to measure blood pH, because blood flow was immediately stopped upon mice euthanasia.

On the other hand, acidic duodenal pH has been reported to alter gene expression in the pancreas of a cystic fibrosis mouse. When the duodenal pH was corrected, either genetically (breeding CFTR-null with gastrin-null mice) or pharmacologically (use of the proton pump inhibitor omeprazole), expression levels of genes measured by quantitative RT-PCR were significantly normalized^19^.

Our work could be considered a hypothesis generator study. Was the blood acidosis detected in a closed corporeal entity in hypoxia a consequence for the compensation of the tissue alkalosis produced in the first instance in the hypoxic tissues? This hypothesis should be further explored since high-throughput molecular assays are playing an ever-growing role in research and into the understanding of the pathobiology of human and animal diseases.

In conclusion, this work found significant differences in pH values between organs at the same cold-ischemia time, and in the same organ at different times in an experimental animal model, although no differences were seen in the RNA quality assessed by its integrity number or absorbance ratios. Our results suggest that pH is high in tissues undergoing ischemia, a finding that has already been reported in previous studies, and the regulation of tissue pH after organ removal may minimize biological stress in human tissue samples. The results from this study suggest that although the RIN is a powerful tool to inspect the ribosomal profile to infer RNA quality from fresh and frozen tissues (and to compare samples RIN values given the same organism/tissue/extraction method), it is not sufficient to predict the integrity of mRNA transcripts and describe the real biological conditions. Therefore, the role of pH as an underlying mechanism of gene expression changes after an ischemic insult warrants further investigation.

## Materials and Methods

Fifty-two mice (Mus musculus) C57BL/6-SPF from the Animal Facility at the CIPE were used. The study was approved by the Ethics Committee in the Use of Animals (CEUA) at Antonio Prudente Foundation (N°049/12). All experiments were performed in accordance with relevant guidelines and regulations. Animals were divided into 5 groups according to the time of tissue cryopreservation. All mice were pathology free (data not shown). The 26 male and 26 female mice were carefully selected in order to have a similar weight (24.89 ± 5.21 gr) and time of life for each gender (104 ± 36 days, Supplementary Fig. S1). The procedure involving the removal of organs was carried out under laminar flow and aseptic conditions through surgical incision by the median line. The material used for the surgery was submerged –between procedures– for 2 minutes in 0.3% (v/v) Endozyme AW plus Apa solution (Ruhof Corporation Biomed Division, NY, USA). The timers were activated for the recording of the different times of tissue cold ischemia exactly upon heart removal when blood flow stopped (0, 15, 30, 45 and 60 minutes). Both lungs, both kidneys and two equal portions of the liver were removed and weighed (Supplementary Fig. S2). Once the organs were weighed, they were divided into two parts, the largest for cryopreservation and a smaller portion (10–30–60 mg of lung, kidney and liver, respectively) for the determination of tissue pH. The organ removed was left out at room temperature until the required time elapsed. Then, pH was measured and the tissue cryopreserved.

### Histopathology

The anatomopathological study was carried out using cryopreserved material. It was placed in Tissue-Tek® O.C.T. Compound (O.C.T. optimal cutting temperature medium, Sakura® Finetek, Torrance, CA, USA). Three μm sections were cut in a −28 °C cryostat. Excess of Tissue-Tek® O.C.T. compound was separated and tissue was saved in the corresponding cryotube for subsequent macromolecules extraction. The histoarchitecture was studied using standard hematoxylin-eosin staining, to assess the morphological changes induced by cold-ischemia.

### 260/280 ratio

The ratio of absorbance at 260 nm and 280 nm (A260/280) was used to assess RNA purity. A ratio of 2.0 or greater is accepted as pure RNA (Supplementary Fig. S3A). A lower ratio may imply the presence of protein, phenol or other contaminants strongly absorbing at or near 280 nm^20^. The effects of pH and ionic strength have shown that the change in the A260/280 ratio is primarily due to a decrease in the absorbance at 280 nm, when the ionic strength or pH is increased. This effect was achieved irrespective of the method of purification. However, the elution method can interfere with the A260/280 ratio. Thus, RNA diluted into distilled-DEPC-treated-water has usually a lower ratio compared with Tris-EDTA pH 8.0.

### 260/230 ratio

This ratio was used as a secondary measure of nucleic acid purity. The A260/230 values for pure nucleic acid are 1.8 or higher (Supplementary Fig. S3B). Where the ratio is lower than expected, it may indicate the presence of contaminants. Nucleic acids absorb at 260 nm. Contaminants like guanidine thiocyanate, phenol, buffer, ethanol, proteins and solvents, used for RNA extraction, absorb at or near 230 nm. Samples with A260/230 value below 1.8 have a significant contamination with organic solvents that may interfere with other experimental approaches, such as microarray experiments, thus interfering with their quality.

### RNA Quality

The cryopreserved tissue of the 312 samples was placed in a mortar with liquid nitrogen (104 kidney, liver and lung portions). The RNA was isolated from up to 30 mg of disaggregated solid frozen tissue, which was homogenized with Precellys®24 (Bertin Technologies, Montigny-le-Bretonneux, France) for 4 cycles (10 seconds at 6500 rpm and 5 minutes in ice), and centrifuged 3 minutes at 13000 rpm. Supernatant was separated for total RNA purification, and pellet was saved at −20 °C for DNA extraction, if necessary. The RNA was purified using RNeasy Mini Qiacube Kit – Animal tissue and cells – Large Samples (QIAGEN, Venlo, Netherlands) on the automated Qiacube (Qiagen, CA, USA). RNA concentration and purity were measured with NanodropTM ND-1000 (Thermo Scientific, DE, USA). RNA integrity was determined with Agilent RNA 6000 Nano chip (Agilent Technologies, Waldbronn, Germany) and measured on Agilent Bioanalyzer-2100 (Agilent Technologies, CA, USA). Agilent Bioanalyzer showed a numerical value (RIN-RNA Integrity Number) which varies from 1 to 10, with 1 referring to degraded RNA and 10 to full RNA^13^. This user-independent RIN is calculated using a software algorithm that contemplates the entire electrophoretic trace of the RNA, including presence or absence of degradation products. The Bioanalyzer software also generates an electropherogram and gel-like image of the 18S and 28S RNA ribosomal ratio. RIN numbers greater than 7 correlate with successful downstream microarray experiments, as manufacture indication (Supplementary Fig. S4).

### RNA concentration

RNA concentration over 0.1 µg/µl was considered the minimum concentration required for sample acceptance (Supplementary Fig. S5).

### pH

The pH was measured at room temperature in technical duplicates for both lungs and kidneys, and for the anterior and posterior portions of the liver (Supplementary Fig. S6). The tissue was manually homogeneized without dilution using a micro pistill^16^. Thus, each animal had twelve different measurements of pH, four for each organ (Dual Channel pH-Ion-meter Accumet Excel-XL25, Thermo Fisher Scientific, CA, USA).

### Statistical Analysis

Statistics were performed using InfoStat software version 2015^21^ together with R version 3.2.3 packages^22, 23^ for model estimation. Mice age comparison by gender was carried out using Wilcoxon rank sum test with continuity correction, since normality assumption between these two groups was not achieved (Supplementary Fig. S1). In order to consider the complete experimental design and technical constraints, independent linear mixed-effects models were estimated. Briefly, the fixed effects were the global mean contribution (µ) of the corresponding dependent output variable; the i-th organ indicator (α_i_ for lung, liver or kidney); the j-th linear (β_j_ = 0, 15, 30, 45, 60 min), quadratic (γ_j_ = β_j_^2^) or third (δ_j_ = β_j_^3^) time degree; the double time by organ interactions terms (α_i_ × β_j_, α_i_ × γ_j_, α_i_ × δ_j_); the k-th gender indicator (θ_k_ for male or female), and the l-th organ side indicator (ρ_l_ for right or left). The random effects were the m-th animal a_m_ ~ N(0, Iσ_a_^2^), i.e. normally distributed with zero mean and identity the variance matrix (I) times, the σ_a_^2^ parameter; the n-th technical replicate within animal effect b_n(m)_ ~ N(0, Iσ_b_^2^), the o-th sample extraction day c_o_ ~ N(0, Iσ_c_^2^), the p-th surgery day d_p_ ~ N(0, Iσ_d_^2^), and the random error function g_(ε)ijklmnop_ which was modeled as independent errors, i. e., g(ε)_ijklmnop_ = I(ε)_ijklmnop_ = ε_ijklmnop_ ~ N(0, Iσ_ε_^2^) or dependent errors, with the lack of heterocedasticity being corrected with a constant variance function grouped by animals, formally, g(ε)_ijklmnop_ = varIdent (~1|Animal)_ijklmnop_. Using the former annotations, the different experimental data measurements (y_ijklmnop_) were modeled starting from the maximal model of equation (1):

1 $$\begin{array}{rcl}{y}_{ijklmnop} & = & \mu +{\alpha }_{i}+{\beta }_{j}+{\gamma }_{j}+{\delta }_{j}+{\alpha }_{i}\times {\beta }_{j}+{\alpha }_{i}\times {\gamma }_{j}+{\alpha }_{i}\times {\delta }_{j}+{\theta }_{k}+{\rho }_{l}\\  &  & +{a}_{m}+{b}_{n(m)}+{c}_{o}+{d}_{p}+g{(\varepsilon )}_{ijklmnop}\end{array}$$

Model selection was carried out using backward elimination over log-likelihood test results, Akaike information criterion (AIC) and Bayes information criterion (BIC) under the parsimony criterion. The corresponding model assumptions were verified using residual vs. predicted values and normal quantile-quantile plots. When required, outlier values were removed and models were estimated again. For the final model assessment, marginal hypothesis test was used with type-III sum of squares for fixed effects. A posteriori analyses included Fisher’s least significant difference (LSD) using Bonferroni adjusted p-values at each time period or main effect. All statistical tests, unless otherwise specified, were two-sided with a cut-off at 0.05 for statistical significance. Equations (2–9) and Supplementary Table S1 show the final adjusted model for pH, RIN, RNA A260/280 (RNA 260/280) and A260/230 (RNA 260/230), RNA concentration (RNA cc) and relative organ weight (ROW) = (organ weight)/(animal weight), lung or kidney relative side weight (RLW or RKW) = (organ side weight)/(animal weight).

2 $$p{H}_{ijklmnop}=\mu +{\alpha }_{i}+{\beta }_{j}+{\gamma }_{j}+{\delta }_{j}+{\alpha }_{i}\times {\beta }_{j}+{\alpha }_{i}\times {\gamma }_{j}+{\alpha }_{i}\times {\delta }_{j}+{a}_{m}+a{(b)}_{mn}+{c}_{o}+{d}_{p}+{\varepsilon }_{ijklmnop}$$

3 $$RI{N}_{ijklmnop}=\mu +{\alpha }_{i}+{\beta }_{j}+{\gamma }_{j}+{\alpha }_{i}\times {\beta }_{j}+{\alpha }_{i}\times {\gamma }_{j}+{c}_{o}+{d}_{p}+g{(\varepsilon )}_{ijklmnop}$$

4 $$RNA\,260/{280}_{ijklmnop}=\mu +{\alpha }_{i}+{a}_{m}+{c}_{o}+{d}_{p}+{\varepsilon }_{ijklmnop}$$

5 $$RNA\,260/{230}_{ijklmnop}=\mu +{\alpha }_{i}+{c}_{o}+g{(\varepsilon )}_{ijklmnop}$$

6 $$RNA\,c{c}_{ijklmnop}=\mu +{\alpha }_{i}+{\beta }_{j}+{\gamma }_{j}+{\delta }_{j}+{\alpha }_{i}\times {\beta }_{j}+{\alpha }_{i}\times {\gamma }_{j}+{\alpha }_{i}\times {\delta }_{j}+{c}_{o}+g{(\varepsilon )}_{ijklmnop}$$

7 $$RO{W}_{ijklmnop}=\mu +{\alpha }_{i}+{\theta }_{k}+{c}_{o}+g{(\varepsilon )}_{ijklmnop}$$

8 $$RL{W}_{ijklmnop}=\mu +{\theta }_{k}+{\rho }_{l}+{c}_{o}+{d}_{p}+{\varepsilon }_{ijklmnop}$$

9 $$RK{W}_{ijklmnop}=\mu +{\rho }_{l}+{a}_{m}+{c}_{o}+{d}_{p}+{\varepsilon }_{ijklmnop}$$

Ischemia time evolution for pH in the three different organs studied is depicted in Fig. 1. Table 1 shows pH and RIN adjusted means with LSD results. Independent time variables, such as RNA ratios and associated relative weight are described in Table 2. Complete model results for the different variables of interest are shown in Supplementary Table S1.

## Electronic supplementary material

Supplementary Data
