# Supplementary material for: Tissue alkalosis in cold-ischemia time
Source: Sci Rep. 2017 Sep 7;7:10867. doi: 10.1038/s41598-017-11284-z (PMC5589730; doi:10.1038/s41598-017-11284-z)
Supplement: Supplementary file 1 — Supplementary Data [file 41598_2017_11284_MOESM1_ESM.docx]

**Tissue alkalosis in cold-ischemia time**

V. Denninghoff^1,2,#,*^, E. H. R. Olivieri^3,4^, C. Fresno^5,#^, A. Uceda, L. D. C. Mota^4^, A. P. M. S. Silva^4^, D. M. Carraro^3,4^, V. R. Martins^3^, M. A. Avagnina^2^, F. A. Soares^1^, A. H. J. Fróes Marques Campos^1,4,*^

^1^Department of Anatomic Pathology, A.C. Camargo Cancer Center, São Paulo, Brazil.

^2^Department of Pathology & Biobank, Center for Medical Education and Clinical Research “Norberto Quirno” (CEMIC), Ciudad Autónoma de Buenos Aires, Argentina.

^3^International Center for Research, A.C.Camargo Cancer Center (CIPE), São Paulo, Brazil.

^4^A.C.Camargo Biobank, A.C.Camargo Cancer Center, São Paulo, Brazil.

^5^Computational Genomics Division, National Institute of Genomic Medicine (INMEGEN), México city, México.

^#^National Scientific and Technical Research Council (CONICET).

*Corresponding authors.

Supplementary Data

The animal age distribution of both 26 females and 26 males was designed to be similar to that depicted in Supplementary Fig. S1, with identical median values at 91.5 days, despite the difference in interquartile range. Our design was confirmed by Wilcoxon’s rank sum test with continuity correction results (p=0.94), i.e. no statistical differences (bias) could be attributed to age gender. Quality control was carried out regarding the relative organ weight, which was defined as the weight ratio between the organ and the mouse model. Two lung samples were excluded from the analysis, since they were defined as structural outliers (Z-Score>5). These samples could have had a systematical weight bias due to technical organ manipulation. Supplementary Fig. S2A shows the filtered relative organ weight boxplots grouped by organ. Note that the highest values belong to the liver, followed by the kidney and lung, with equation (7) results showing a p<0.0001 for the organ effect (Supplementary Table S1). To further assess structural outlier samples, we inspected the relative lung and kidney weight according to their laterality (right or left). Three additional structural outliers were found on the lung left-side and were excluded from the analysis. Filtered results are shown in Supplementary Fig. S2B. As expected, the left lung has a lower relative weight according to equation (8) results (p<0.0001, Supplementary Table S1), since it only has two lobules, instead of the three of its right counterpart, which is located next to the heart. In addition, the relative kidney weight in equation (9) was also significant for side effect (p<0.0001, Supplementary Table S1), which was probably due to model power, since there is no anatomical evidence for that.

The RNA absorbance ratio raw measurements for 260/280 and 260/230 for the different times, grouped by organs, are shown in Supplementary Fig. S3A and S3B, respectively. Interestingly, and as far as equation (4) and (5) results, RNA absorbance ratios were constant and only differed by the organ effect in both cases (p<0.0001, Supplementary Table S1). Fisher’s least significant difference between organs showed that for both mean RNA ratios (Supplementary Fig. S3C and S3-D) the kidney outperforms (A group) both liver and lung (B group). The dashed line in every panel represents the minimum quality control threshold for sample inclusion in the analysis as described in the experimental design section. The RIN second-order time evolution linear mixed model results of equation (3) are shown in Supplementary Fig. S4A, Table 1 and S1. As it may be seen with the complementary Fisher’s LSD results in Supplementary Fig. S4B, the integrity achieved in this study outperforms the minimum acceptance threshold of RIN=7 for every organ. Despite the fact that the kidney had the clearest pronounced descendant pattern (RIN=10 to RIN=9, approximately), all organs RINs median was above nine. Although our statistical power could adjust a RIN second-order organ polynomial, in biological terms samples could be considered of the highest quality. No evidence of possible variability RIN bias due to intra-chip or inter-chip variability was found in the three RNA Nano chips used, where the coefficient of variance was 6.5, 7.43 and 5.94% (typically the range is 5-10%). Finally, it is worth mentioning that RNA concentration data were adjusted using a third-degree polynomial model, as depicted in Supplementary Table S1 and Fig. S5. However, the authors postulate this behavior could be associated with technical differences, rather than with ischemia effect itself, as this variable only refers to the proper sample preparation which, in all cases, was above the minimum quality threshold required per sample (0.1 µg/µl). The NanoDrop ND-1000 spectrophotometer (Fisher Thermo, Wilmington, DE, USA) is accurately to quantify RNA in the 2ng/ul to 3000 ng/ul. According to Aranda et al., RNA concentration measurements on the NanoDrop ND-1000 spectrophotometer results in the smallest coefficient of variance (Cv) variation when compared with RiboGreen and the Agilent Nano and Pico kits, but with large variability below 2,5 ng/ul measurements (Cv values > 20%)^23^. Our experimental design is using Nanodrop at an acceptable RNA concentration working point, thus, the variability of Fig. S5 is not affected.

As the aim of this study was focused on tissue pH changes by ischemia time, pH was measured using two technical replicates for each sample (N° 624). Three structural outliers were removed from the analysis, since they failed to follow the pH scatter plot for technical replicates of Supplementary Fig. S6A. Note that the black line indicates the identity function - error free measurement - where our data had an R^2^=0.89 between the two replicates. In addition, the boxplot of the combined pH replicate values grouped by organs is shown in Supplementary Fig. S6B. The lung had the highest median pH, followed by the kidney and liver. However, equation (2) results revealed that no significant differences were found by organ effect (p=0.16, Supplementary Table S1). These differences are associated with their time interaction (Fig. 1, Table 1 and S1).

Supplementary
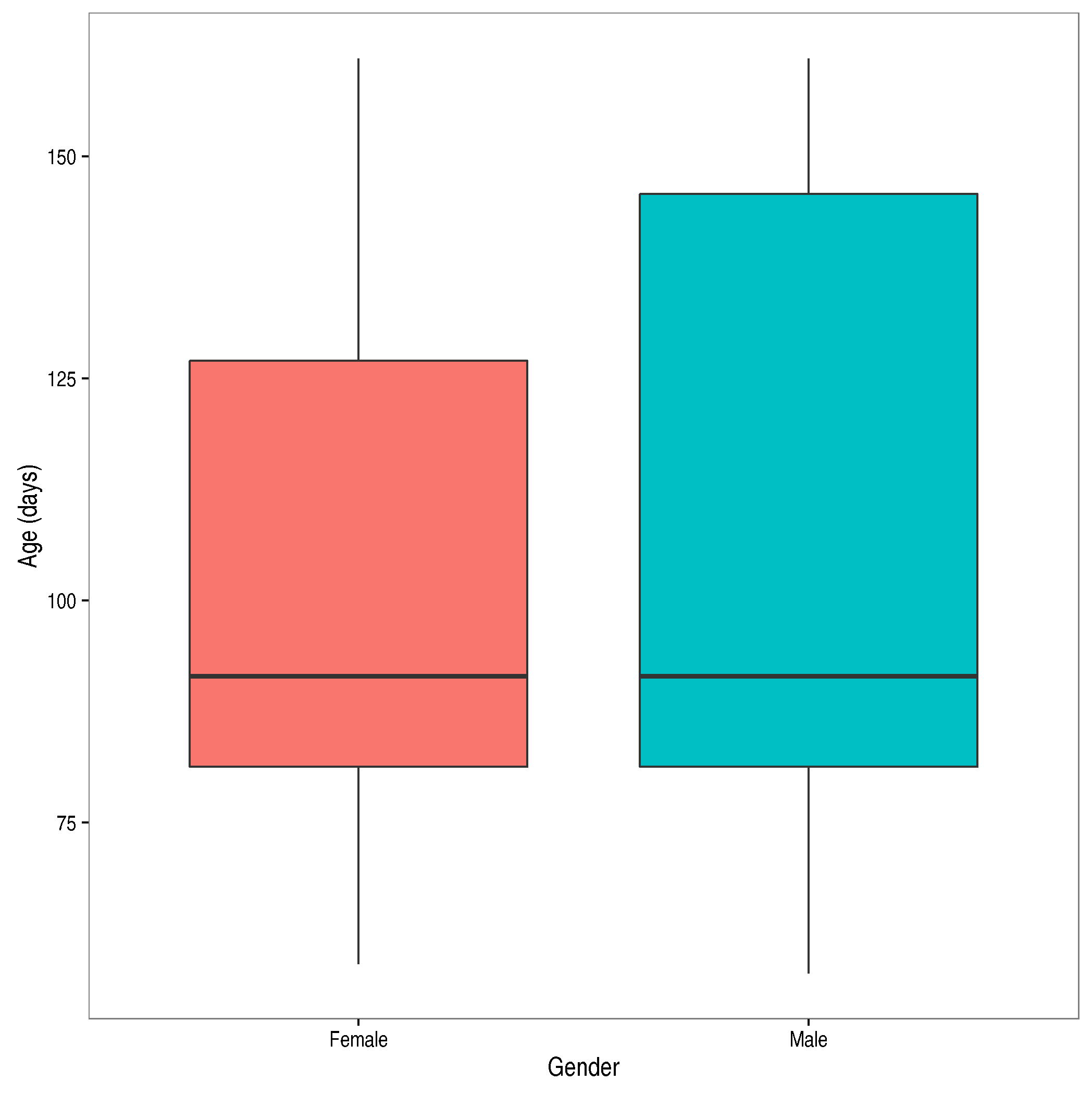
Fig. S1. Animal age. The animal age boxplots are grouped by gender (26 females and 26 males). Interestingly, the age range was designed to be similar to that depicted by identical median values at 91.5 days, despite the difference in interquartile range. The results were confirmed by Wilcoxon’s rank sum test with continuity correction results (p=0.94).

Supplementary
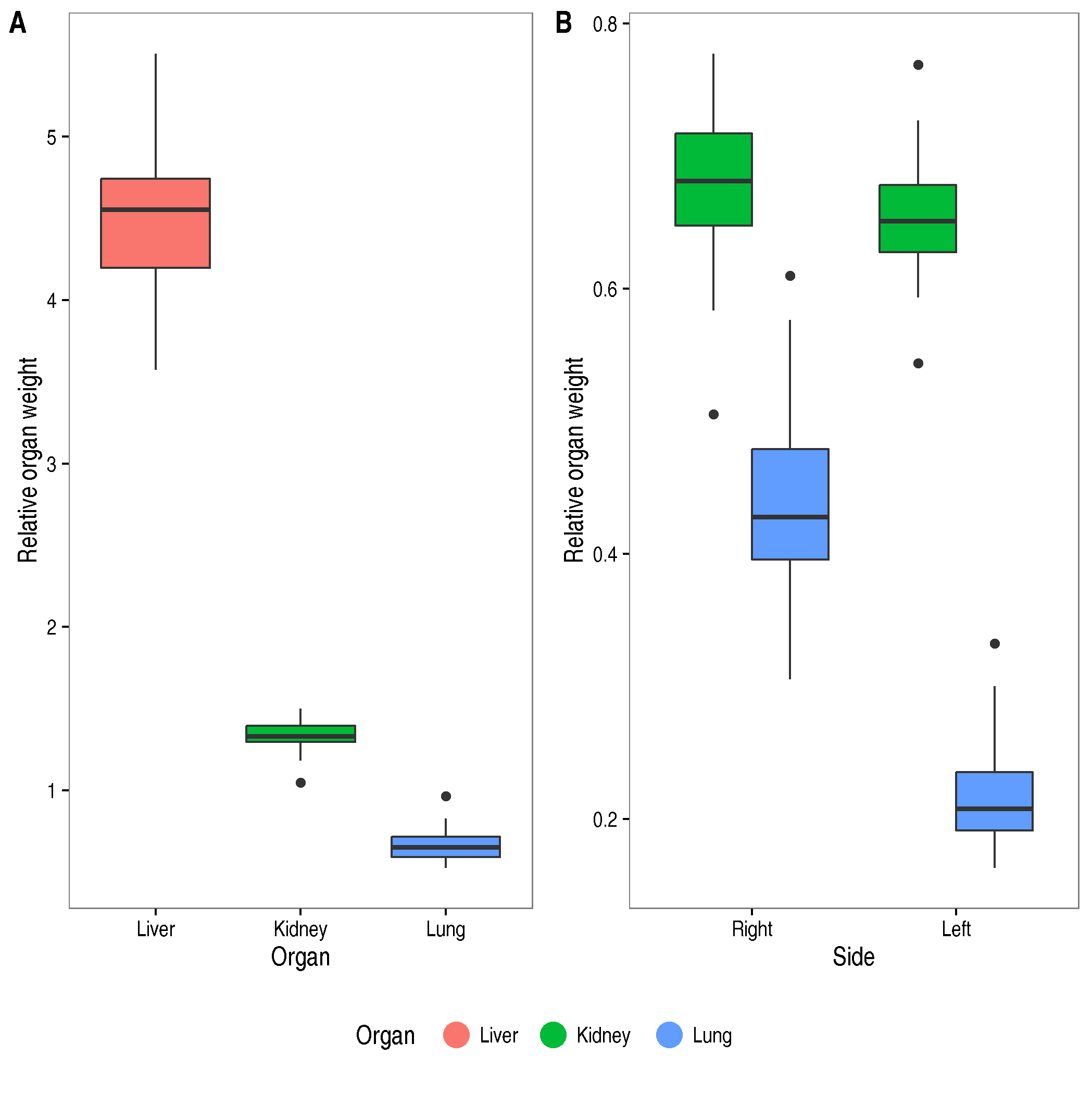
Fig. S2. Relative organ weight. A) Relative organ weight boxplots are grouped by organ. Note that the highest values belong to the liver, followed by the kidney and lung, with equation (7) having a p<0.0001 for the organ effect (Supplementary Table S1). B) Only lung and kidney relative weight boxplots are shown according to their side (right or left). As expected, the left lung has a lower relative weight according to equation (8) results (p<0.0001, Supplementary Table S1), since it only has two lobules, instead of three as in the case of its right counterpart. In addition, the kidney relative weight in equation (9) had also a significant side effect (p<0.0001, Supplementary Table S1) which could be associated with model power, since there is no anatomical evidence for that.

**Supplementary
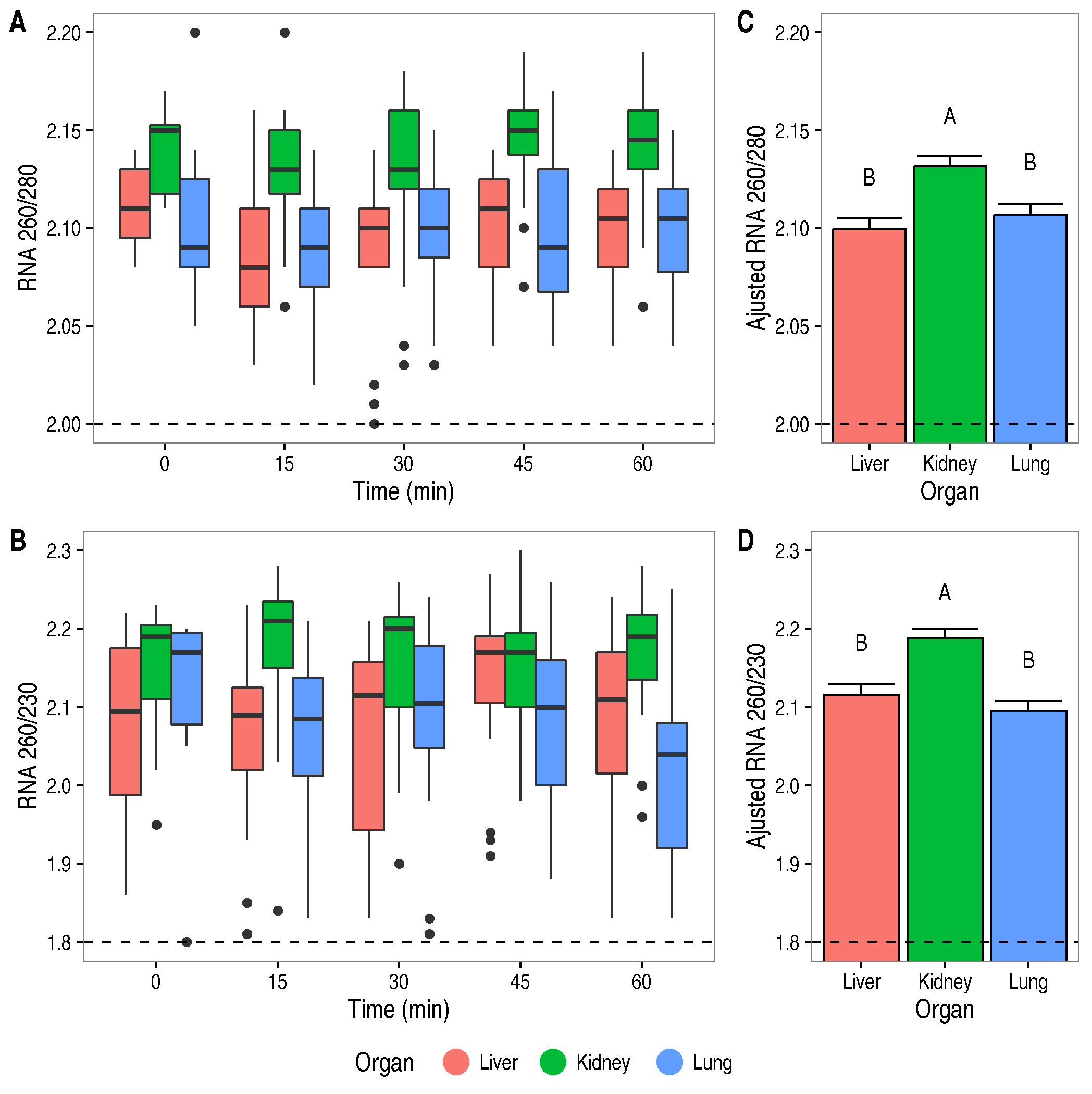
Fig. S3. RNA absorbance ratios.** Panels A and B show RNA absorbance boxplot ratios of 260nm/280nm (RNA 260/280) and 260nm/230nm (RNA 260/230), respectively. These are grouped by organs according to the different times under analysis. Interestingly, and as evidenced by equation (4) and (5), RNA absorbance ratios results were constant and only differed by the organ effect in both cases (p<0.0001, Supplementary Table S1). Fisher’s least significant difference between organs is presented as mean ± standard error estimation in panel C) and D) for 260/280 and 260/230 RNA ratios, respectively. Superscript letters indicate significant difference (p<0.05) using Bonferroni adjusted p-values. Note that for both mean RNA ratios the kidney (A group) outperforms both liver and lung (B group). The dashed line in every panel represents the minimum quality control threshold for sample inclusion in the analysis.


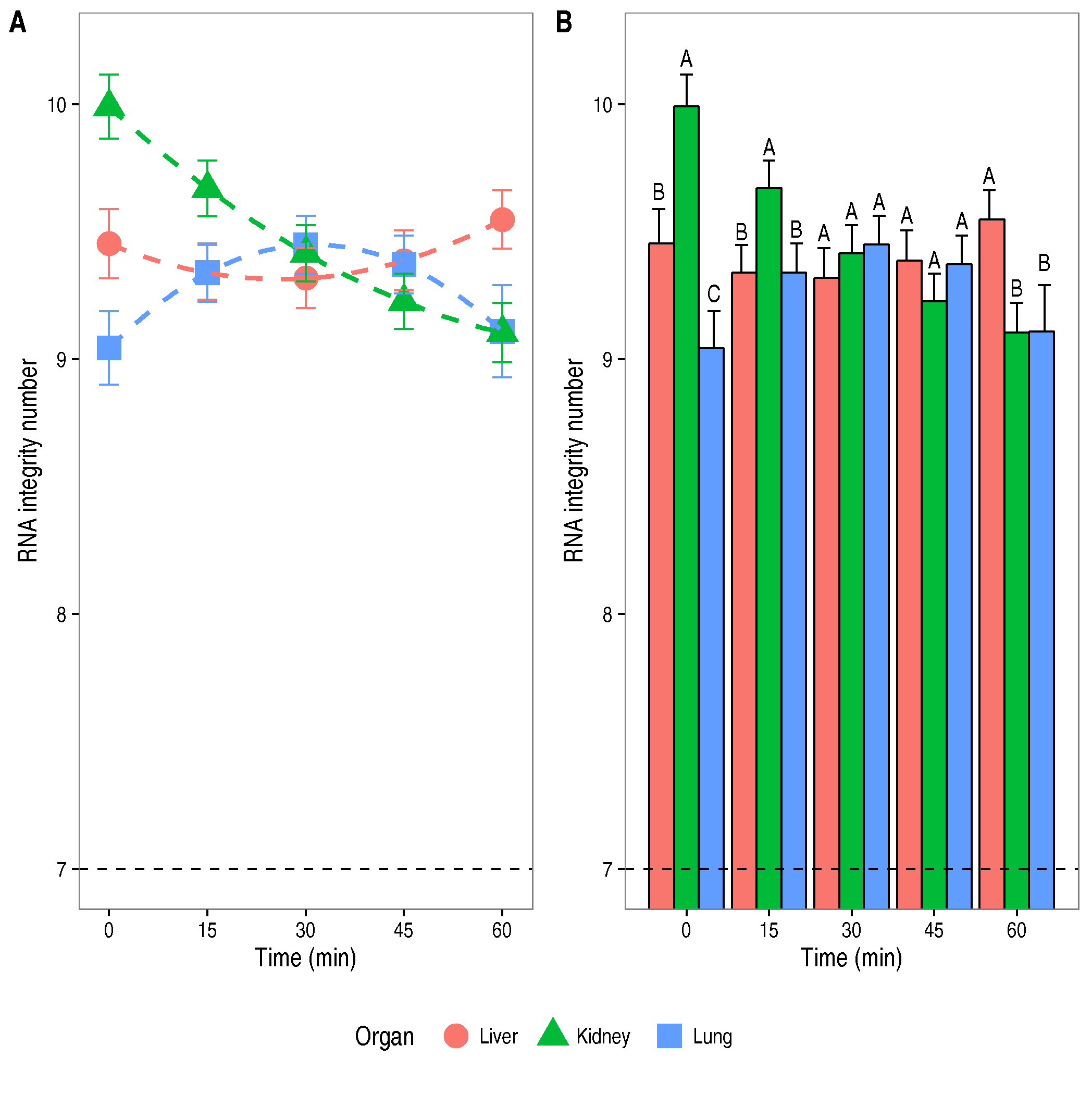


Supplementary Fig. S4. RNA integrity number time evolution. A) Organs second-degree adjusted time polynomial linear mixed model results of equation (3) in dashed lines. B) Fisher’s least significant difference between organs mean at each data point. Superscript letters indicate a significant difference (p<0.05) using Bonferroni adjusted p-values. In both panels, results are presented as the mean ± standard error estimation, and the dashed line at seven represents the minimum quality control threshold for sample inclusion in the analysis.


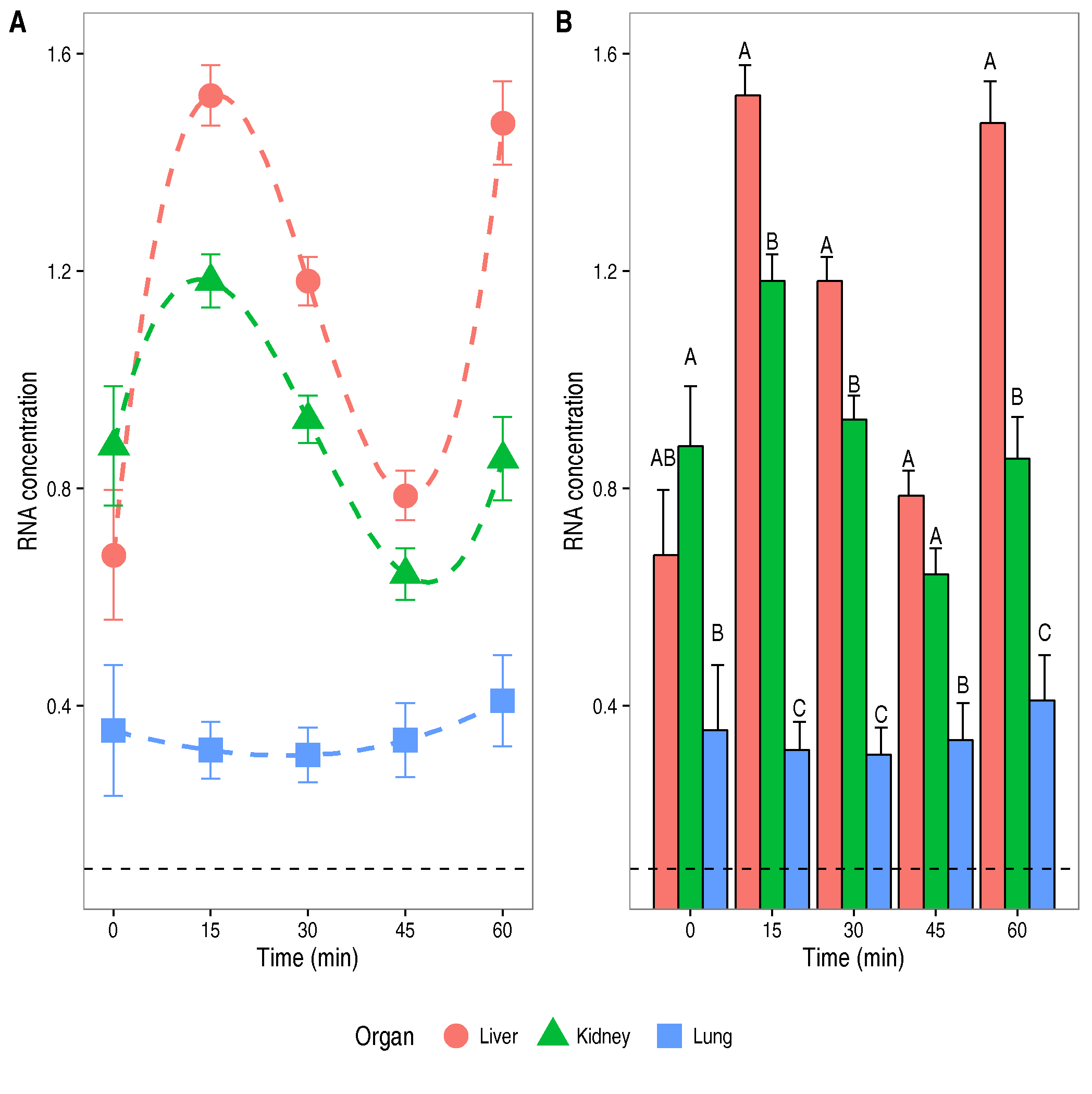


Supplementary Fig. S5. RNA concentration time evolution. A) Organs third-degree adjusted time polynomial linear mixed model results of equation (6) in dashed lines. B) Fisher’s least significant difference between organs mean at each data point. Superscript letters indicate a significant difference (p<0.05) using Bonferroni adjusted p-values. In both panels, results are presented as the mean ± standard error estimation, and the dashed line at 0.1 µg/µl represents the minimum quality control threshold for sample inclusion in the analysis.


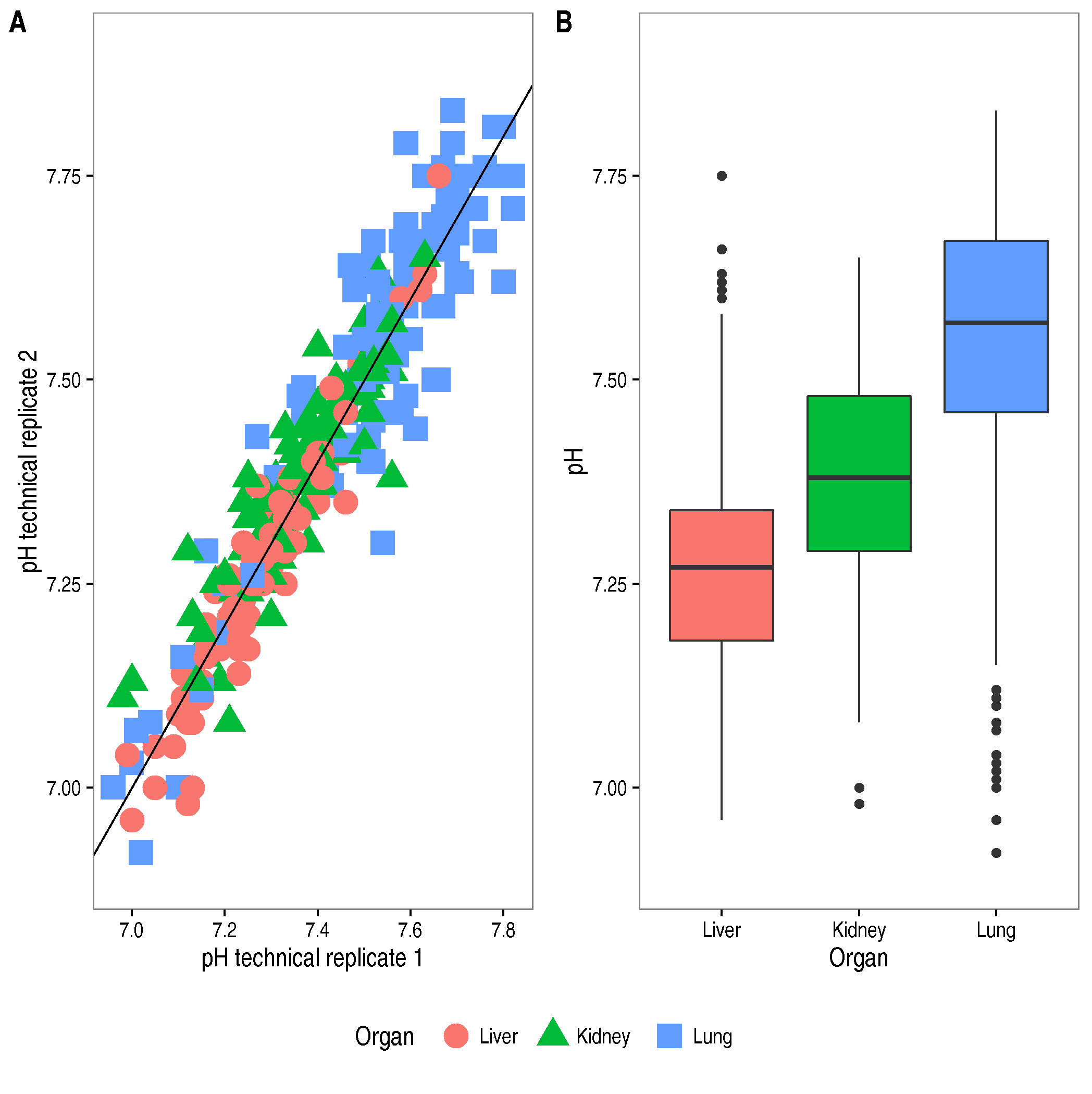


Supplementary Fig. S6. pH quality control. A) pH technical replicate comparison for the different organs and times. The black line indicates the identity function, i.e. an error-free replicate. B) Boxplot of the combined pH replicate values grouped by organs. Note that the lung has the highest median pH, followed by the kidney and liver. However, equation (2) results showed no significant difference by organ effect (p=0.16, Supplementary Table S1), but median pH is associated with its time interaction (Fig. 1, Tables 1 and Supplementary Fig. S1).

Supplementary Table S1: Model results for the different variables of interest.

| Variable | | | pH | RIN | RNA | | | ROW | RLW | RKW |
| --- | --- | --- | --- | --- | --- | --- | --- | --- | --- | --- |
|  |  |  |  |  | 260/280 | 260/230 | cc |  |  |  |
|  | N | | 559 | 281 | 279 | 246 | 280 | 114 | 88 | 102 |
| Fixed effects | µ | Mean | *** | *** | *** | *** | *** | *** | *** | *** |
|  | α | Organ | 0.16 | *** | *** | *** | * | *** | - | - |
|  | β | Time | *** | 0.6 | - | - | *** | - | - | - |
|  | γ | Time^2^ | ** | 0.79 | - | - | *** | - | - | - |
|  | δ | Time^3^ | * | - | - | - | *** | - | - | - |
|  | α x β | Interac- tions | *** | *** | - | - | *** | - | - | - |
|  | α x γ |  | *** | ** | - | - | *** | - | - | - |
|  | α x δ |  | * | - | - | - | *** | - | - | - |
|  | θ | Gender | - | - | - | - | - | *** | *** | - |
|  | ρ | Side | - | - | - | - | - | - | *** | *** |
| Random effects | σ_a_ | Animal | 1.20σ_ε_ | - | 0.19σ_ε_ | - | - | - | - | 0.75σ_ε_ |
|  | σ_b(a)_ | Technical replicate | 1.74σ_ε_ | - | - | - | - | - | - | - |
|  | σ_c_ | Extraction | 0.08σ_ε_ | 0.92σ_ε_ | 0.51σ_ε_ | 0.48σ_ε_ | 0.06σ_ε_ | 0.13σ_ε_ | 0.29σ_ε_ | - |
|  | σ_d_ | Surgery | 1.04σ_ε_ | 0.10σ_ε_ | 0.11σ_ε_ | - | - | - | 0.30σ_ε_ | - |
|  | ε or g(ε) | Error structure | ε | g(ε) | ε | g(ε) | g(ε) | g(ε) | ε | ε |

N: number of samples included in the model. RIN: RNA Integrity Number; RNA 260/280 and RNA 260/230 are the RNA absorbance ratios at 260nm/280nm and 260nm/230nm, respectively; RNA cc: RNA concentration; ROW: Relative organ weight, i.e. its weight normalized by the animal total weight; RLW and RKW are the lung and kidney relative side weight, respectively. Significance convention used for marginal type-III sum of squares for fixed effects is *: p<0.01; **: p<0.001; ***: p<0.0001, with the effect being excluded at the model selection stage. Random effects standard deviations (σ_a_, σ_b(a)_, σ_c_, σ_d_) are expressed regarding the residual (σ_ε_). ε: independent random error structure, i. e., ε ~ N(0, Iσ_a_^2^). g(ε): lack of heteroscedasticity was corrected using a constant variance function grouped by animals.
